# Ovary Preservation for Cervical Clear Cell Adenocarcinoma in an 8‐Year‐Old Girl: A Novel Approach

**DOI:** 10.1155/crog/6722243

**Published:** 2026-07-16

**Authors:** Lin Wang, Weiguo Hu, Jinqiu Zhang, Angang Sun, Yu Chen, Huijian Li, Shaojie Zhao, Dengxin Zhang, Yue Wu, Qi Chen, Yanfang Gu, Min Zhao

**Affiliations:** ^1^ Department of Gynaecological Oncology, Wuxi Maternity and Child Health Hospital, Jiangnan University, Wuxi, China, jiangnan.edu.cn; ^2^ Department of Obstetrics, Gynaecology and Reproductive Science, Faculty of Medical and Health Sciences, The University of Auckland, Auckland, New Zealand, auckland.ac.nz; ^3^ Department of Gynaecological Oncology, Hospital of Obstetrics and Gynaecology, Fudan University, Shanghai, China, fudan.edu.cn; ^4^ Department of Pathology, Wuxi Maternity and Child Health Hospital, Jiangnan University, Wuxi, China, jiangnan.edu.cn; ^5^ Department of Reproductive Endocrinology, Wuxi Maternity and Child Health Hospital, Jiangnan University, Wuxi, China, jiangnan.edu.cn; ^6^ Department of Gynaecology, Wuxi Maternity and Child Health Hospital, Jiangnan University, Wuxi, China, jiangnan.edu.cn; ^7^ Department of Anesthesia, Wuxi Maternity and Child Health Hospital, Jiangnan University, Wuxi, China, jiangnan.edu.cn

## Abstract

Cervical clear cell adenocarcinoma is a rare malignancy in adolescents and young girls. Since the prescription of diethylstilbestrol (DES) was banned in 1971, only 11 cases have been reported in girls under 10 years in the literature. However, treatment with ovary preservation has not been reported. Here, we report an 8‐year‐old prepubertal girl without a history of exposure to DES who was diagnosed with cervical clear cell adenocarcinoma. Hysterectomy and unilateral oophorectomy were performed. The remaining ovary was transposed to the left iliac fossa to preserve ovarian function for the future. Additionally, ovarian cortical tissue from the removed ovary was cryopreserved. Chemotherapy, including paclitaxel and carboplatin, was started after surgery for four cycles via an implantable venous access port. Additionally, genetic analysis did not identify any known gene mutations in the tumour tissue.

## 1. Introduction

Cervical cancer is predominantly diagnosed in adult women aged 35 years and older, although recent trends suggest a gradual shift toward younger age at diagnosis, possibly due to increased HPV screening and awareness [1]. A study reported that among women under 40 years, 78% of diagnosed cases occurred in those aged 30 to 39 years [2]. In contrast, cervical cancer is extremely rare in the adolescent population. Most reported cases in the literature occur in adolescents older than 15 years, with an incidence rate of approximately 0.15 per 100,000 [2]. Unfortunately, current cervical cancer screening methods, such as Pap smears and HPV testing, are not routinely applied in children and adolescents, limiting early detection in this group [2].

Several studies have documented cervical cancer in adolescents aged 15 to 19 years, with a limited case number, typically associated with high‐risk HPV infection [2–5]. However, clear cell adenocarcinoma is exceedingly rare in the adolescent population and is a special type of human papillomavirus (HPV)‐independent cervical cancer. In contrast, cervical clear cell adenocarcinoma was historically linked to uterine exposure to diethylstilbestrol (DES). A recent systematic review included 29 studies and reported 35 cases with cervical clear cell adenocarcinoma without direct exposure to DES in adolescents under 15 years since 1976 [6]. In that systematic review study, fewer than 11 cases were reported to be under 10 years. Two additional studies with cervical clear cell adenocarcinoma have recently been reported in adolescents aged 14 years [7, 8].

The treatment options for cervical clear cell adenocarcinoma typically mirror those for other types of cervical cancer and are stage dependent. A combination of surgery, including radical hysterectomy, chemotherapy and radiation therapy, is commonly recommended. However, for adolescent girls, maintaining hormonal function, overall long‐term health and the potential for future fertility are critical considerations. Ovary preservation technology, such as ovarian tissue cryopreservation, offers significant long‐term health benefits, including sustained natural hormone production, a reduced risk of cardiovascular disease, improved cognitive function and an enhanced quality of life [9].

In this study, we present a case of a rare cervical clear cell adenocarcinoma in an 8‐year‐old prepubertal girl who had no history of direct or indirect exposure to DES and who underwent ovary preservation surgery in the management.

## 2. Case Presentation

An 8‐year‐old girl presented with irregular vaginal bleeding that had persisted for 6 months, initially with a small amount of bleeding. A urinary tract infection was initially suspected and diagnosed based on the presence of white blood cells in the urine. The girl was then treated with traditional Chinese herbs to control infection, which temporarily alleviated the symptoms. However, the irregular vaginal bleeding persisted for 5 months. A colposcopy was suggested but was not performed at that time. In the gynaecological clinic, due to the presence of redness on the inner side of the labia minora, vaginitis was suspected and treated. To exclude precocious puberty, serum levels of sex hormones, including follicle‐stimulating hormone (FSH), luteinizing hormone (LH), anti‐Müllerian hormone (AMH) and *β*‐human chorionic gonadotropin (*β*‐hCG), were measured, and all values of sex hormones were within normal ranges for her age (FSH: 23 IU/L, LH: 1.47 IU/L, AMH: 2.10 ng/mL and HCG negative). The vaginal bleeding did not appear to have cured, and the girl then revisited our gynaecological clinic.

**Figure 1:**
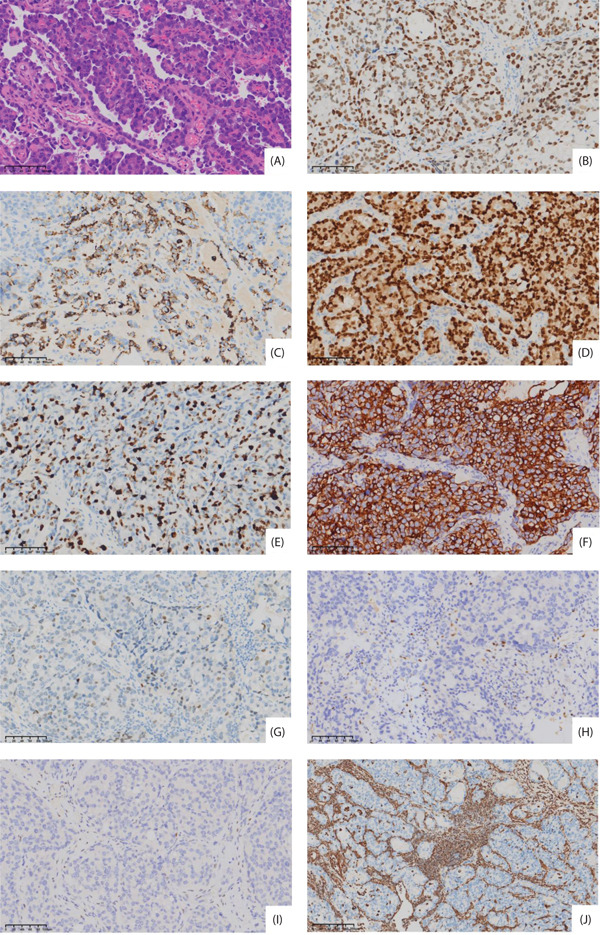
(A) Haematoxylin and eosin staining showing an infiltration of tumour cells with a tubular‐cystic growth pattern in the cervix (Bar: 100 *μ*m). (B) Showing the positivity of hepatocyte nuclear factor 1‐beta (HNF‐1*β*) in tumour cells (Bar: 100 *μ*m). (C) Showing the positivity of Napsin A in tumour cells (Bar: 100 *μ*m). (D) Showing the positivity of PAX 8 in tumour cells (Bar: 100 *μ*m). (E) Showing the positivity of Ki‐67 in tumour cells, with more than 50% (Bar: 100 *μ*m). (F) Showing the positivity of pan‐cytokeratin in tumour cells (Bar: 100 *μ*m). (G) Showing the weak positivity (one +) of p53 in tumour cells (Bar: 100 *μ*m). (H) Showing the negative of oestrogen receptor in tumour cells (Bar: 100 *μ*m). (I) Showing the negative of progesterone receptors in tumour cells (Bar: 100 *μ*m). (J) Showing the negative of vimentin in tumour cells (Bar: 100 *μ*m).

Physical and gynaecological examination revealed a body weight of 55 kg and a height of 138 cm (BMI: 28.88 kg/m^2^), with pubertal development classified as “Tanner stage I”. There was no abnormal finding in the vagina with slight redness in the labia minora. No abdominal pain or palpable mass, or inguinal lymphadenopathy was noted. Colposcopy examination found a sized 2 × 1 × 1 cm irregular cervical mass arising from the upper lip of the cervix. The histology of cervical tissues from colposcopy suggested the possibility of clear cell adenocarcinoma, stage IB2 (Figure 1A), with positive staining of hepatocyte nuclear factor 1‐beta (Figure 1B), Napsin A (Figure 1C) and paired box gene 8 (PAX 8) (Figure 1D). Additionally, histology revealed Ki‐67 positivity at 50% (Figure 1E), pan‐cytokeratin positivity (Figure 1F) and weak p53 positivity (+) (Figure 1G) in tumour tissue. However, staining for oestrogen or progesterone receptors and vimentin was negative in the tumour tissue (Figures 1H–J). Postoperative histology confirmed no lymph node metastasis and stromal invasion limited to the superficial third, with no evidence of lymphovascular space invasion (LVSI).

The patient was then immediately referred to the gynaecological oncology clinic. Peripheral tumour markers were assessed. Details were cancer antigen (CA) 125: 10.2 U/L, CA153: 5.6 U/L, CA199: 4.8 U/L, CA72‐4: 1.3 U/L, carcinoembryonic antigen (CEA): 0.82 ng/mL, squamous cell carcinoma antigen (SCCA): 0.86 ng/mL, human epididymis protein 4 (HE4): 23.8 pmol/L and alpha‐fetoprotein (AFP): 1.1 ng/mL. Pelvic magnetic resonance imaging (MRI) revealed no ovarian abnormalities, but the uterine structure was poorly visualized. No enlarged lymph nodes (lymphadenopathy) and pelvic effusion were detected in the pelvic cavity.

The patient′s medical history was unremarkable, with no history of medication use in either father or mother, including during pregnancy. Her elder brother was born with LGA (large for gestational age) 13 years ago. There were no family members previously diagnosed with cancer in either the mother or the father. The mother had not undergone an HPV test before pregnancy. However, HPV‐negative status in the mother was reported in the last 2 years. Neither mother nor father smokes.

A recent study suggested that bilateral salpingo‐oophorectomy does not improve long‐term survival, and ovarian preservation is oncologically safe in early‐stage cervical cancer, including adenocarcinoma, and should be routinely considered, particularly for premenopausal women without evidence of ovarian invasion [10]. After a multidisciplinary team meeting involving gynaecological oncologists, gynaecologists, paediatricians, pathologists, anaesthetists and charge nurses, the decision was made to include a laparoscopic radical hysterectomy with Type C1, bilateral salpingectomy, pelvic lymph node clearance and a unilateral oophorectomy (right side) in the case management plan. The left ovary was reserved and transposed to the left iliac fossa to maintain ovarian function and minimize exposure to postoperative adjuvant therapy. Additionally, ovarian cortical tissue from the removed right ovary was cryopreserved. Following radical hysterectomy, histopathological examination revealed a residual tumour measuring 0.2 × 0.2 cm, confined to the cervix, with no evidence of vaginal invasion. The discrepancy between the clinically estimated tumour size and the final pathological size reflects a prior diagnostic biopsy, with only minimal residual tumour identified in the hysterectomy specimen. The intraoperative findings were consistent with the preoperative MRI findings.

No inherited genetic changes and mutated genes that are associated with cervical cancer development were found in tumour tissues by molecular investigation.

Two weeks after the operation, the patient was discharged without any complications. Cervical clear cell adenocarcinoma is an aggressive, rare histological subtype with a high propensity for early recurrence and distant metastasis. Additionally, studies reported that receiving multiple cycles of adjuvant chemotherapy is associated with significantly better disease‐free survival and overall survival [11]. To target occult micrometastases and improve long‐term outcomes, a regimen of Paclitaxel (210 mg, Day 1) and Carboplatin (500 mg, Day 2) based on body weight was selected for four cycles, administered 1 month postsurgery, with an implantable venous access port. The first cycle has now been completed as of the time of this report.

## 3. Discussion

The causes of cervical clear cell adenocarcinoma are not well understood. However, uterine exposure to DES is a significant contributor to this condition. Although the age at diagnosis of cervical clear cell adenocarcinoma is between 15 and 31 years, the median age at diagnosis in women without DES exposure was between 50 and 53 years [12, 13]. In the literature, since the prescription of DES was banned in 1971, only 11 cases have reported cervical clear cell adenocarcinoma in adolescent girls under 10 years without direct DES exposure (Table 1). However, recently, a case of an 8‐year‐old girl whose grandmother was exposed to DES during pregnancy was reported [22], suggesting a possibility of multigeneration effects in humans.

**Table 1:** The literature review of cervical clear adenocarcinoma in girls under 10 years old.

Age at diagnosis (years)	Tumour size (cm)	Adjuvant therapies	Follow‐up period (months)	Outcomes	References
1	1.5 × 1.9 × 2.3	Not performed	8	Alive	Arora et al. [14]
6	2 × 2 × 1	Not performed	12	Alive	Ahrens et al. [15]
6	< 2	Not performed	8	Alive	Lester et al. [16]
6	1.5 × 2	Chemotherapy (docetaxel and oxaliplatin)	28	Alive	Su et al. [17]
6	1.4	Not performed	12	Alive	Abu‐Rustum et al. [18]
7	4 × 6	Radiation and chemotherapy (Taxol/carboplatin)	35	Alive	Wesolowski et al. [19]
7	> 4	Palliative irradiation	335	Alive	Noller et al. [20]
8	3.2 × 3.5 × 2	Vaginal brachytherapy	24	Alive	Hutten et al. [21]
8	2	Not performed	10	Alive	Abu‐Rustum et al. [18]
8	1.7 × 0.7 × 0.7	Radiation	120	Alive	Gaspari et al. [22]
10	Not mentioned	Chemotherapy (carboplatin and paclitaxel)	> 3	Died	Asri et al. [23]

The most common symptom of cervical clear cell adenocarcinoma is vaginal bleeding, which is easily misdiagnosed in adolescent girls or prepubertal girls. Alongside a very small number of case reports in the literature, accurate diagnosis of cervical clear cell adenocarcinoma in adolescent girls is a challenge, even in adolescents who have prolonged vaginal bleeding. To date, only two cases have reported the computed tomography (CT) scan and MRI findings of cervical tumours in a 13‐year‐old [24] and 15‐year‐old girls [25]. Due to the limitations of vaginal examination in adolescents, those studies then suggested promptly considering diagnostic imaging to avoid misdiagnosis. In our case, we did not find a cervical tumour by MRI. This could be because the tumour size was 2 × 1 cm in our case, smaller than that in the previous two studies (4 × 3 cm or 7 × 4 cm).

Cervical clear cell adenocarcinoma is associated with a poor prognosis, even in adults. There were no standardized guidelines for the treatment of cervical clear cell adenocarcinoma, but the treatment options are normally referred to other histology types and stages of cervical cancer. A radical hysterectomy with pelvic lymphadenectomy constitutes a standard treatment [26]. For the early stage of cervical clear cell adenocarcinoma, chemoradiation or radiation is recommended after surgery to help reduce the chance of recurrence. In our case, in addition to hysterectomy, four cycles of chemotherapy with paclitaxel and carboplatin were decided, and due to the higher BMI, an implantable venous port was placed for chemotherapy.

In the literature, several patients had transposition of the ovary for fertility preservation before chemoradiation, and none of them had recurrence or death to date. Maintaining hormonal function, overall long‐term health and the potential for future fertility is critical for young girls. Like a previous study involving unilateral oophorectomy and preservation of the other ovary [12], in our case, the left ovary was transposed to the left iliac fossa to maintain ovarian function during surgery, and ovarian cortical tissue from the removed right ovary was additionally cryopreserved. This technology was confirmed by the American Society for Reproductive Medicine in 2019. Although we do not yet have follow‐up data related to disease progression in our case, another study [12] reported a 42‐month disease‐free survival rate after ovary preservation.

The poor progression of cervical clear cell adenocarcinoma, including recurrence, has been reported in adults. Although there is no overall data available on disease progression and recurrence of cervical clear cell adenocarcinoma in adolescents, the survival rate reported to date is relatively high. According to the literature search, 10 cases of cervical clear cell adenocarcinoma in individuals under 10 years of age were alive, at least within 8 months, and in some cases, even longer, up to 335 months after treatment (Table 1), depending on the period of follow‐up. Although only one case died 3 months after six cycles of chemotherapy because of recurrence. Additionally, a recent study using the SEER database reported that younger girls under 14 years had a better prognosis than older ones, above 15 years, although this study did not separate the tumour sites (vagina or cervix) and tumour types (clear cell adenocarcinoma, embryonal rhabdomyosarcoma, or yolk sac tumours) [27]. That study also reported a 5‐year overall survival rate of 89% for clear cell adenocarcinoma. Clearly, long‐term follow‐up is important for generating data on overall survival.

Genetic changes or mutations can contribute to the development of cervical clear cell adenocarcinoma, such as the mutation of the CMTM5 gene, which is an inherited genetic mutation increasing the risk of cancer. However, our molecular testing for specific genetic markers did not identify any mutated genes associated with the development of cervical cancer in tumour tissues.

In conclusion, here we report a rare case of cervical clear cell adenocarcinoma in an 8‐year‐old girl. In addition to a hysterectomy, we also performed an ovary preservation surgery by transposition of one side of the ovary to the left iliac fossa. Molecular analysis did not identify any known gene mutations in the tumour tissue, suggesting genetic changes may not be associated with the development of cervical clear cell adenocarcinoma.

## Author Contributions

All authors were involved in drafting, editing and approving the manuscript for publication. M.Z. and Q.C.: conceived and designed the study. L.W., H.L. and Y.W.: data collection. W.H., M.Z., Y.C., S.Z., D.Z. and Y.G.: clinical management discussion. J.Z. and A.S.: histology analysis.

## Funding

No funding was received for this manuscript.

## Consent

Publication of this case report was obtained with the consent of the patient′s guardian.

## Conflicts of Interest

The authors declare no conflicts of interest.

## Data Availability

The datasets used and/or analyzed during the current study are available from the corresponding authors on reasonable request.
